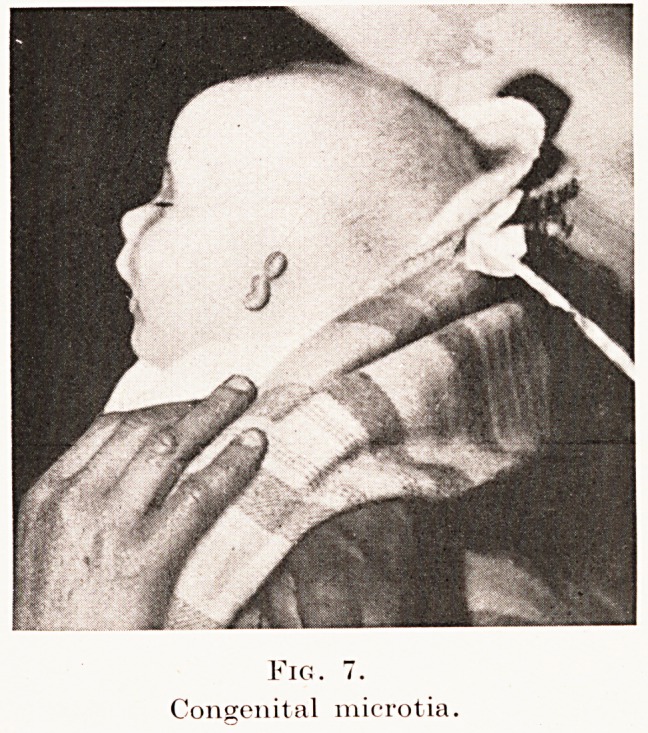# Congenital Abnormalities of the External Ear

**Published:** 1931

**Authors:** E. Watson-Williams

**Affiliations:** Surgeon in Charge, Ear, Nose and Throat Department, Bristol Royal Infirmary


PLATE XVI.

FIG. 1.

FIG. 2.

Congenital " Lop-ear."

FIG. 3.

" Faun's Ear."

FIG. 4.
Double accessory auricle: note also the deformity of the pinna.

### PLATE XVII.

Fig. 5.
Uvoid accessory auricle.

Fig. 6.
Congenital microtia.

Fig. 7.
Congenital microtia.

### CONGENITAL ABNORMALITIES OF THE EXTERNAL EAR.

BY

E. Watson-Williams, M.C.,

*Surgeon in Charge, Ear, Nose and Throat Department, Bristol Royal Infirmary.*

---

Congenital deformities of the external ear are not very uncommon. They may consist merely of a departure from the usual shape sufficiently pronounced to attract attention; for example, absence of the lobule, which is said to be relatively common among the mentally defective and criminals, and on the Continent classified as a "stigma of degeneration." Rarer types are the "lop-ear," in which the pinna or its upper part hangs down over the meatus (Figs. 1 and 2); and the "faun's ear," in which the tubercle of the helix is erect and conspicuous. (Fig. 3.) The last variety represents a condition normal in fœtal life, and which, by its relation to certain simian ears, attracted the attention of Darwin. All these types tend to be symmetrical.

Errors of excess produce the most frequent deformity, the accessory auricle. The common type is a simple tubercle situated immediately in front of the tragus. It tends to run in families, to affect boys, and is often unilateral. I have seen an example in which such a deformity occurred on the right side in father, son and grandson. Much more rarely one finds two accessory auricles on the same side, as shown in

Fig. 4. The brother of this little boy had a single accessory auricle on the corresponding side. A rare form of accessory auricle is the pedunculated uvoid lobule, found below the ear on the side of the neck. (Fig. 5.) It resembles the extra lobule normal in goats, and is interesting from its frequent representation in classical statues of satyrs. These lobules are commonly bilateral.

Errors of defect are less common. The pinna may be merely small, and when this is unilateral a curious effect is produced, as in the celebrated example from Devizes. In the more extreme cases of congenital microtia the external ear is represented merely by one or two small protuberances ; while a shallow dimple indicates the position of the meatus. (Figs. 6, 7.) The bony meatus in these cases is often absent, and the middle ear may participate in the abnormality ; not seldom the labyrinth and even the eighth nerve show defects of development. For this reason it is generally useless to attempt surgical restoration of the meatus, though there is enough of the middle-ear tract to give rise to mastoiditis. Rarely such a condition is bilateral, and causes deaf-mutism.

An aural fistula results from imperfect closure of the first visceral cleft. Just within the opening of the meatus a small orifice opens on the floor, and may communicate with a branchial cyst of some volume overlying the posterior part of the parotid ; sometimes attention is attracted by the discovery of mucoid discharge from the ear. Surgical removal is quite feasible. A still more rare condition is a persistent cleft, running down from the ear between tragus and antitragus into the side of the neck.

## Figures and Tables

**Fig. 1. Fig. 2. f1:**
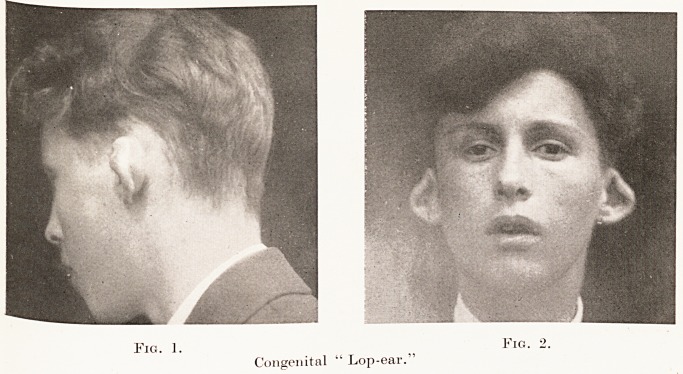


**Fig. 3. f2:**
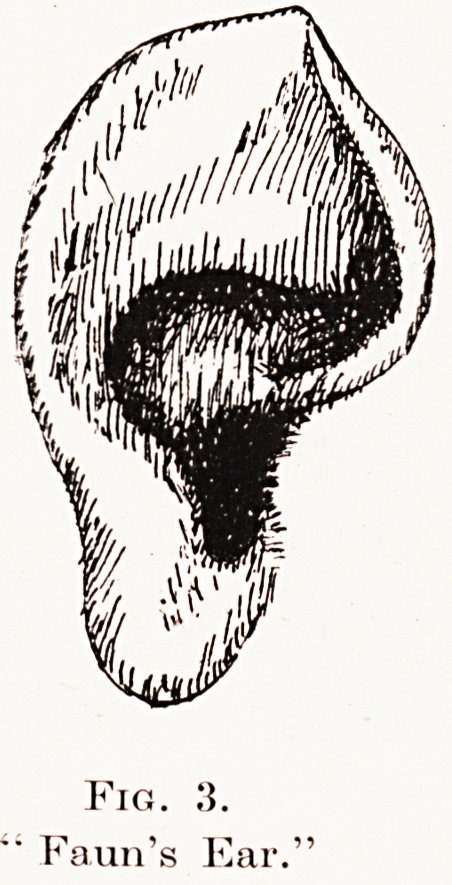


**Fig. 4. f3:**
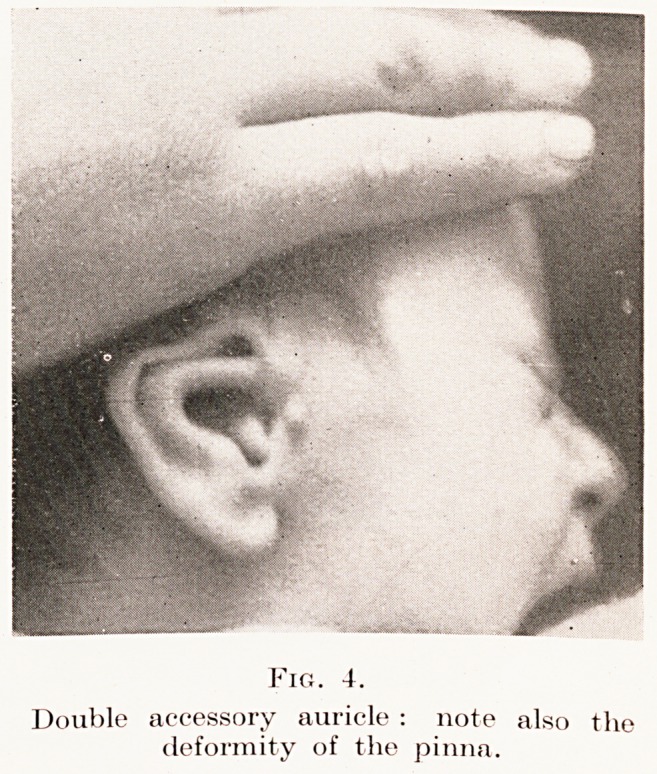


**Fig. 5. f4:**
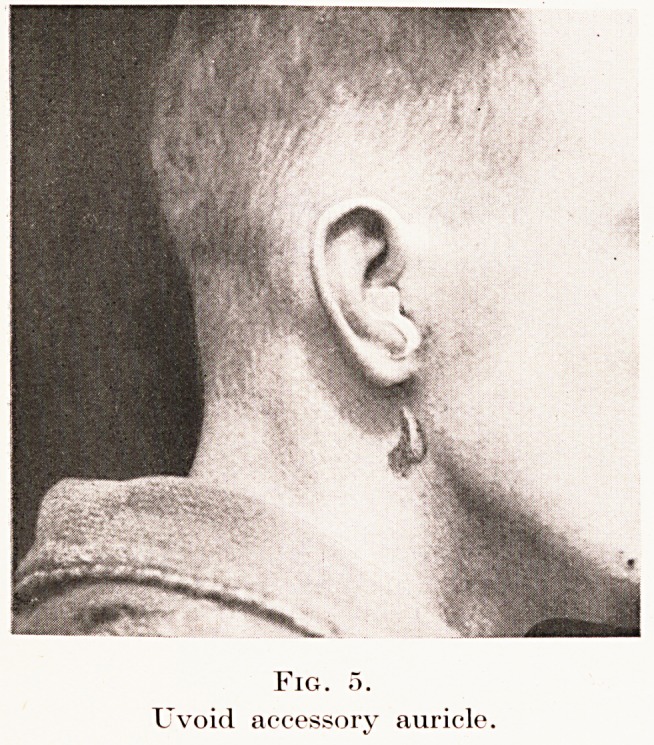


**Fig. 6. f5:**
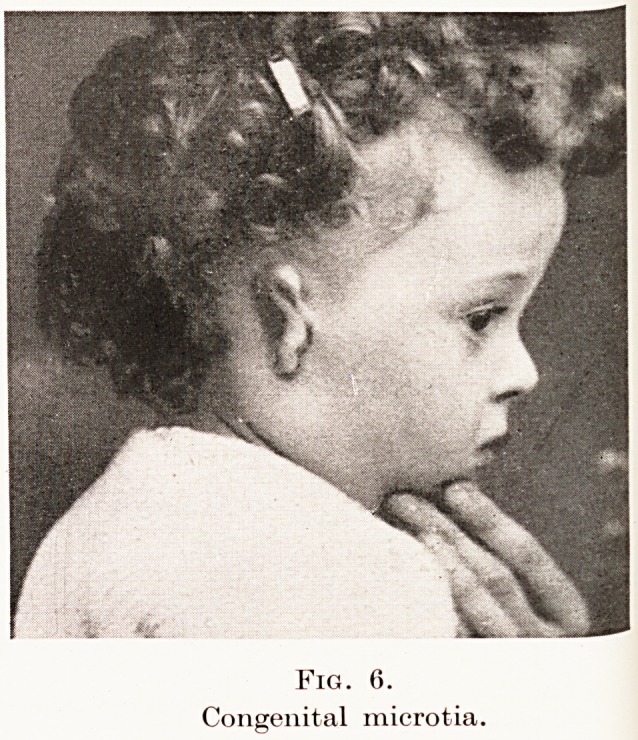


**Fig. 7. f6:**